# Skeletal dysplasia-like syndromes in wild giraffe

**DOI:** 10.1186/s13104-020-05403-9

**Published:** 2020-12-30

**Authors:** Michael Butler Brown, Emma Wells

**Affiliations:** 1 Giraffe Conservation Foundation, Eros, PO Box 86099, Windhoek, Namibia; 2 Smithsonian National Zoo and Conservation Biology Institute, Conservation Ecology Center, 1500 Remount Rd, Front Royal, VA 22630 USA; 3 Department of Biological Sciences, Dartmouth College, Hanover, NH 03755 USA

**Keywords:** Giraffe, Skeletal dysplasia, Disproportionate dwarfism

## Abstract

**Objective:**

Skeletal dysplasias, cartilaginous or skeletal disorders that sometimes result in abnormal bone development, are seldom reported in free-ranging wild animals. Here, we use photogrammetry and comparative morphometric analyses to describe cases of abnormal appendicular skeletal proportions of free-ranging giraffe in two geographically distinct taxa: a Nubian giraffe (*Giraffa camelopardalis camelopardalis)* in Murchison Falls National Park, Uganda and an Angolan giraffe (*Giraffa giraffa* angolensis) on a private farm in central Namibia.

**Results:**

These giraffe exhibited extremely shortened radius and metacarpal bones relative to other similarly aged giraffe. Both giraffe survived to at least subadult life stage. This report documents rare occurrences of these apparent skeletal dysplasias in free-ranging wild animals and the first records in giraffe.

## Introduction

Skeletal dysplasias broadly refer to cartilaginous or skeletal disorders that may result in abnormalities in bone development. These developmental aberrations are sometimes characterized by shortened and irregularly proportioned appendicular skeletal anatomy, resulting in what is vernacularly described as disproportionate dwarfism [10]. Skeletal dysplasias can be caused by a diverse suite of molecular etiologies and can manifest in different forms including micromelia (shortening of the entire limb), rhizomelia (shortening of the femur), mesomelia (shortening of the radius, ulna, tibia, and fibula) [14]. Forms of skeletal dysplasias have been described in a wide range of captive and domestic taxa, including dogs [13], cows [1], pigs [9], rats [18], and common marmosets [3]. However, observations of wild animals with forms of skeletal dysplasia are rare, with notable records of a red deer in Scotland with chondrydysplasia [16] and a male Asian elephant with described disproportionate dwarfism in Uda Walwe National Park (NP) in southern Sri Lanka [7, 19]. Here, we used digital photogrammetry to characterize skeletal dysplasia-like syndromes in two wild giraffe observed during population surveys of geographically distinct taxa—a Nubian giraffe (*Giraffa camelopardalis camelopardalis)* in Murchison Falls NP, Uganda and an Angolan giraffe (*Giraffa giraffa* angolensis) on a private farm in central Namibia. We applied morphometric analyses to compare cervical vertebrae and appendicular skeletal measurements of these two cases to the dimensions of giraffe of different age/sex classes in the Murchison Falls NP population.

## Main text

### Methods

We conducted standard photographic surveys of the giraffe population in Murchison Falls NP, Uganda in association with ongoing research and monitoring programmes developed to examine population dynamics [5]. From July 2014 until March 2019, we regularly surveyed the park at four-month intervals corresponding approximately with seasonal transition periods (March, July, December). During these surveys, we systematically drove a series of fixed routes comprising the road network over the entire extent of the park where giraffe are known to exist. When giraffe were encountered, we photographed the perpendicular lateral view of each individual’s right side and identified every individual giraffe using their unique, unchanging coat patterns in association with WILDID, a specialized pattern recognition software package [2, 8]. In addition to photographing all encountered giraffe, we recorded the spatial coordinates of each observation, sexed each individual, and estimated its age class (calf: 0–12 months; subadult female: 1–3 years; subadult male: 1–6 years; adult female: > 3 years; adult male > 6 years) based on a suite of diagnostic features including body size, limb proportions and secondary sex characteristics (see [17]. In Namibia, we conducted targeted surveys of individual properties using similar survey techniques to establish baseline giraffe population estimates for these areas. Photographic survey methods were strictly non-invasive, were approved by an Institutional Animal Care and Use Committee (IACUC).

**Fig. 1:**
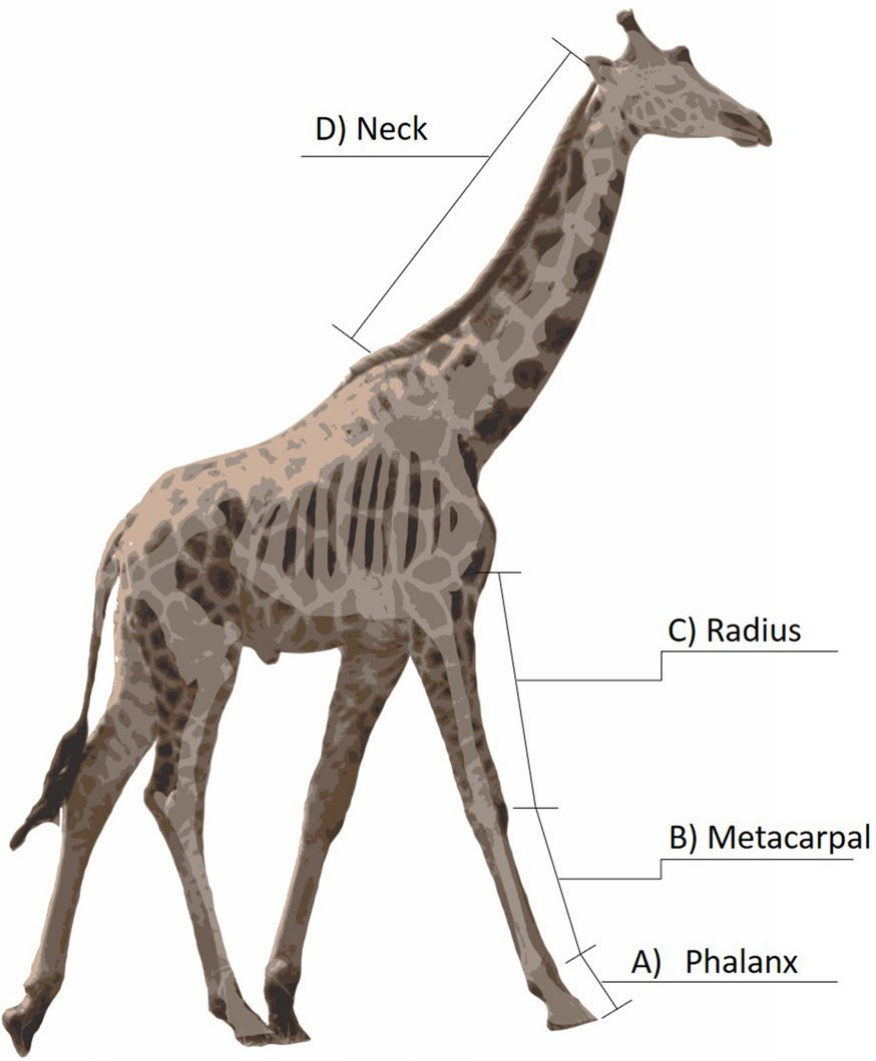
Diagram of diagnostic morphometrics for digital photogrammetry

To better assess age classes and to noninvasively collect morphometric data, we also employed a photogrammetry technique initially designed for measuring African elephant [15] and subsequently adopted for giraffe [11]. This technique uses a laser range finder to measure the distance to features of interest, forming a relationship with digital pixels in the image and actual size of the focal feature thereby allowing for the accurate measurement of giraffe morphological characteristics. To ensure precise relationships between digital pixel size and actual metric units, we first created reference curves for each designated lens focal length by photographing an object of a known size with a digital camera (Canon 7D Mark II body with Canon Ultrasonic IS 100–400 mm lens and Canon 5D Mark II body with a Canon ef 100–400 mm 1:4.5–5.6 L IS lens. Canon U.S.A., Inc., Melville, New York) at 10 m intervals from 10 to 150 m. Since the pixel to centimeter ratio increases linearly with distance to the photographed object, we determined the linear relationship of the number of pixels in a digital image per centimeter of the object photographed over the range of distances photographed using linear regressions for each specific focal length (Fig. in Appendix 1). In the field, when each giraffe was photographed, we used a laser rangefinder (Bushnell Scout Arc 1000, Bushnell Outdoor Products, 8500 Marshall Drive, Lenexa Kansas 66214) to measure the distance of the camera to the giraffe to the nearest 0.1 m. For the corresponding image, we then measured the length in pixels of each of the focal morphological features in the image editing software GIMPv2.8 (GNU Image Manipulation Program, GIMP Development Team, http://www.gimp.org). When possible, we measured the following features:

- Phalanx from the end of the distal phalanx to the approximate end of the lateral proximal sesamoid (Fig. 1a)
- Metacarpal (canon) bone as the lateral proximal sesamoid to the ulnar carpal bone (Fig. 1b).
- Radius as the ulnar carpal bone to the lateral epicondyle (Fig. 1c).
- Neck from approximately the C7/T1 vertebrae to the atlanto-occipital joint (Fig. 1d).

Using the derived camera body specific linear relationships between distance to the giraffe and the associated pixel size at a given focal length extracted from embedded Exif files of the digital image, we calculated the estimated dimensions to the nearest centimeter for each of the featured morphological traits.

To compare morphometric data across age classes, we created a reference morphometric database from the central survey database of all recorded giraffe observations. All encounters from the database were filtered to include only images with the recorded distance data and focal length associated with our pre-calculated calibration curves (100 mm, 200 mm, 300 mm, 400 mm). We then visually inspected the remaining photographs to exclude images in which vegetation and body position obscured potential leg and neck measurements. All reference anatomical measurements were conducted on these resulting images.

Since giraffe exhibit considerable sexual dimorphism as adults, we grouped the measurements of all giraffe according to age classes (adult, subadult, and calf) and partitioned adult giraffe measurements by sex. To evaluate morphological differences of the observed dysplastic giraffe from recorded giraffe of known age classes, we calculated the 95% confidence interval for each measurement for each age/sex class and compared to the observed measurements of each focal dysplastic giraffe to population-level subadult means using one -sample t-tests.

### Results

**Fig. 2:**
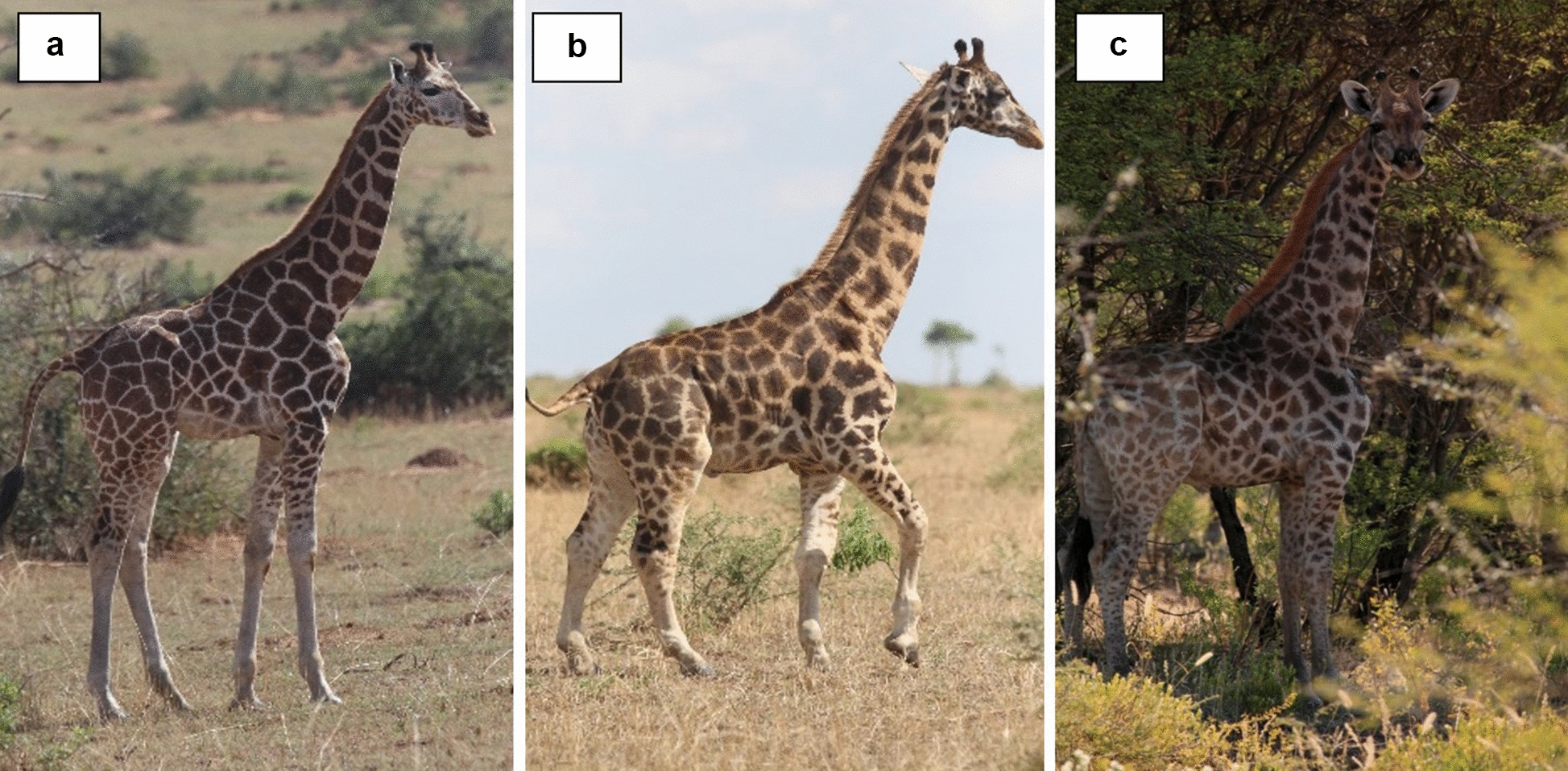
Lateral photographs of giraffe. **a** A typical subadult male giraffe in Murchison Falls National Park, Uganda. **b** A subadult male exhibiting skeletal dysplasia-like syndrome in Murchison Falls National Park, Uganda. **c** A subadult male exhibiting skeletal dysplasia like syndrome on a private farm in Namibia

On 15 December 2015, we first observed a male Nubian giraffe calf in Murchison Falls NP, Uganda with apparently disproportionate limb dimensions relative to torso and neck. We next observed this male nearly one year later on 2 December 2016 and again on 17 March 2017. All subsequent accompanying photographs, measurements, descriptions, and videos of the focal subadult male are associated with the 17 March 2017 observation when the giraffe was known to be at least 15 months of age (Fig. 2b). We observed and photographed a second subadult male giraffe with apparent disproportionate anatomy on a private farm in central Namibia on 10 May 2018, during which time we took photographs and measurements (Fig. 2c). According to the landowner, this giraffe was born in 2014. We observed the Namibian giraffe again on 29 July 2020. No other giraffe were noted with similar morphological abnormalities in either population surveyed.

**Fig. 3:**
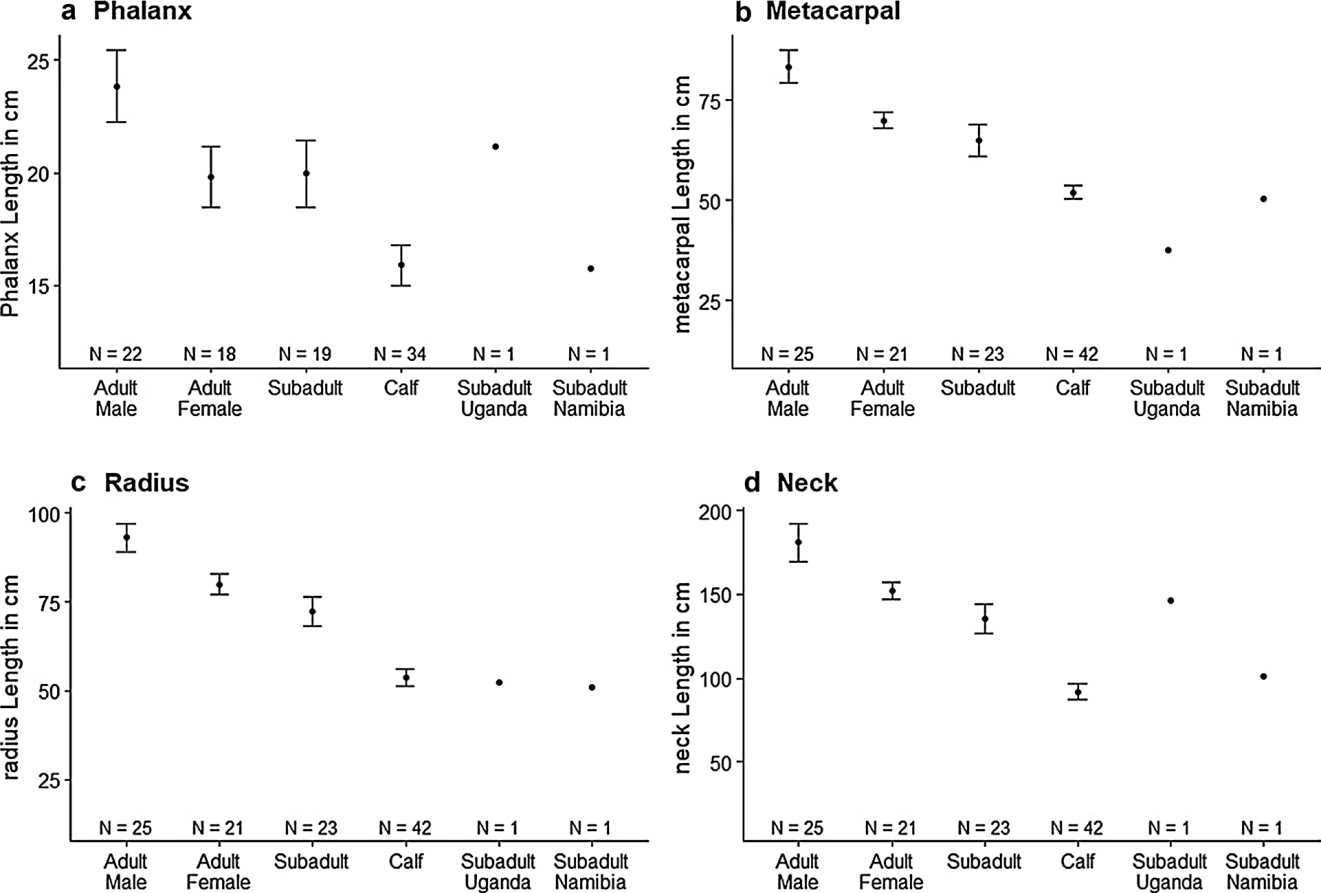
Summary of morphometric analyses for key anatomical features across age/sex classes. Samples sizes for each measurement are enumerated in the corresponding columns. Both the Ugandan and the Namibian focal giraffe comparisons were made with one measurement. Error bars represent 95% confidence intervals around mean values

Morphometric comparisons from photogrammetry measurements indicated that both giraffe with abnormalities had skeletal proportions that differed significantly from population level measurements of subadults (Fig. 3). The Ugandan giraffe exhibited a phalanx length (21.2 cm) consistent with the reference measurements of subadult giraffe at the population level (20.0 cm, 3.30 SD) (t_18_ = − 1.63, p = 0.12), but the Namibian giraffe exhibited a relatively shortened phalanx measurement (15.8 cm) for a subadult giraffe (t_18_ = 5.54, p < 0.01) (Fig. 3a). Both the Ugandan giraffe (37.6 cm) (t_22_ = 13.78, p < 0.01) and the Namibian giraffe (50.5 cm) (t_22_ = 7.31, p < 0.01) exhibited metacarpal dimensions shorter than the population mean for subadults (65.1 cm, 9.57 SD) with the Ugandan giraffe exhibiting an extreme example (Fig. 3b). Both the Ugandan giraffe (52.35 cm) (t_22_ = 9.43, p < 0.01) and the Namibian giraffe (50.89 cm) (t_22_ = − 4.16, p < 0.01) exhibited a radius length shorter than the population mean for subadults (72.17 cm, 10.06 SD) (Fig. 3c). The neck length of the Ugandan giraffe (146.13 cm) (t_22_ = − 2.41, p = 0.02) was greater than the population mean of subadult giraffe (135.21 cm, 21.62 SD) and the Namibian giraffe (101.15 cm) (t_22_ = 7.56, p < 0.01) was shorter (Fig. 3d).

### Discussion

Using digital photogrammetry techniques, we performed comparative morphometric analyses to describe skeletal-dysplasia-like syndromes in two wild giraffe from different taxa and demonstrated that the skeletal dimensions of these dysplastic giraffe are not consistent with the population measurements of giraffe in similar age classes. In both the Ugandan and Namibian giraffe, these conditions were characterized by shortened metacarpal and radius bones. However, these giraffe exhibited shortened fore-limbs to varied degrees, and exhibited different neck lengths, so it is uncertain if the etiology of these skeletal aberrations is consistent across the two presentations.

Although seldomly observed in wild animals, cases of skeletal dysplasia in captive animals have been associated with inbreeding and a lack of genetic diversity [3]. The Murchison Falls NP giraffe population is currently estimated to be > 1350 adult individuals, but it experienced a well-documented bottleneck in the late 1980s, with the population declining to ~ 78 individuals at its nadir [5]. Despite this documented bottleneck event, earlier genetic work on this population suggested relatively low inbreeding estimates [4]. Notably, no other giraffe in these systems exhibited similar skeletal dysplasias during this study period.

Evidence of the potential fitness consequences of similar syndromes in wild animals is lacking, although skeletal dysplasias in some captive animal populations have been associated with lower survival rates [18]. Across giraffe populations, mortality rates are typically highest in calves, with estimated mortality rates during a giraffe’s first year as high as 66% in some populations [12]. Both of the giraffe with observed skeletal dysplasias were older than one year of age, indicating survival past this critical life stage, although the predation rate in Murchison Falls NP is relatively low (M. Brown, pers. comm.) and predators are excluded from the private ranch in Namibia (E. Wells, pers. comm.). Notably, however, despite the high giraffe encounter rates during surveys in Murchison Falls NP and the relatively high subadult/adult survival in the park [6], the Ugandan giraffe has not been observed during surveys since May 2017. The last reported observation of the Namibian giraffe was in July of 2020. Limited mobility caused by shorter leg dimension might make these giraffe more susceptible to predation, even in the subadult/adult life stages. Anecdotal video evidence of giraffe movement in Namibia suggest that this giraffe experience difficulty in movement, with a limping gait (see Additional file 1). Additionally, given that both observed giraffe with skeletal dysplasia were male, successful mounting for breeding seems physically improbable, suggesting the inability to transfer any potential genes associated with this condition.

Here, we report the first documented cases of skeletal dysplasia in two geographically distinct giraffe taxa. These records represent rare cases of these skeletal aberrations in free-ranging wild animals. Additionally, our regular population monitoring provides systematic survey protocols to evaluate survival of these uncommon occurrences, providing an opportunity for deeper insights into the ecology of skeletal dysplasia in wild animals. These skeletal abnormalities are seldom observed in the wild, so systematic monitoring of known individuals and populations where they exist can offer understandings and of the emergence and ecology of these rare phenotypes.

## Limitations

This study was conducted largely on two opportunistic observations of wild giraffe encountered in the field. As such, we are limited by the sample size of individuals and potential imprecision in the laser photogrammetry techniques and are yet unable to conduct longitudinal studies on these individuals. Additionally, we have not yet conducted any genetic analyses to evaluate genetic diversity in the source populations or potential genetic etiologies of the conditions.

### Supplementary Information

**Additional file 1.** A video the focal giraffe in Namibia filmed in July 2020. Note the abnormal gait and limited mobility

## Figures and Tables

**Fig. 4:**
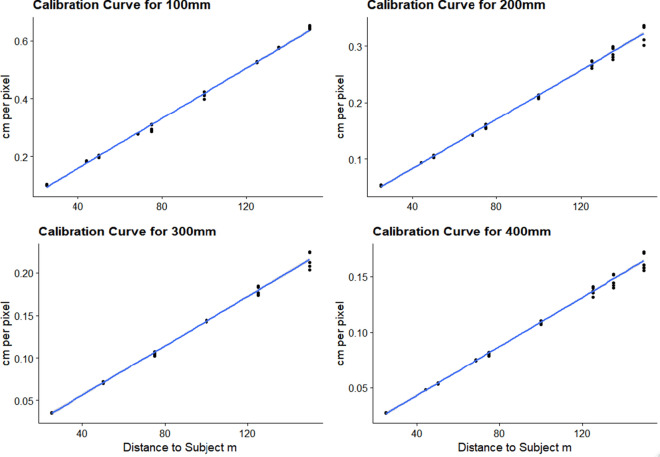
Sample photogrammetry calibration curve for the Canon 7d MarkII body with Canon Ultrasonic IS 100–400 mm lens at multiple focal lengths

## Data Availability

Data are available from the authors upon reasonable request.

## Appendix 1

See Fig. 4.

## Abbreviations

IACUC: Institutional Animal Care and Use Committee
NP: National Park
